# Supplementary material for: Analysis of PPARγ Signaling Activity in Psoriasis
Source: Int J Mol Sci. 2021 Aug 10;22(16):8603. doi: 10.3390/ijms22168603 (PMC8395241; doi:10.3390/ijms22168603)
Supplement: Supplementary file 1 [file ijms-22-08603-s001.zip › Supplemental materials_Analysis of PPARg signaling activity in psoriasis/Pathway models/Models images and html files/PPARG inhibits phenotypes assotiated with psoriasis/133004.html]

heart disease


# Disease heart disease

|  |  |
| --- | --- |
| URN | urn:agi-meshdis:Heart%20Diseases |
| Total Entities | 37 |
| Connectivity | 6181 |
| Name | heart disease |

---

|  |  |
| --- | --- |
| ChildConcepts | heart failure |
|  | ischemic heart disease |
|  | myocardial disease |
|  | valvular heart disease |
|  | cardiac arrhythmia |
|  | cardiac allograft rejection |
|  | cardiotoxicity |
|  | cardiac injury |
|  | congenital heart disease |
|  | electrocardiographic abnormality |
|  | endocardial disease |
|  | myocardial abscess |
|  | intracardiac thrombus |
|  | cardiac hemosiderosis |
|  | cardiac aneurysm |
|  | cardiac neoplasm |
|  | pericardial disease |
|  | cardiac atrophy |
|  | acyanotic congenital heart disease |
|  | rare cardiac disease |
|  | extrasystole |
|  | high cardiac output |
|  | cardiac death |
|  | ventricular dysfunction |
|  | major adverse cardiac event |
|  | cardiac obstruction |
|  | atrial obstruction |
|  | heart calcification |
|  | cardiac remodeling |
|  | cardiac stress |
|  | myocardial stiffness |
|  | cardiac invasion |
|  | cardiac contractile dysfunction |
|  | cardiac autonomic dysfunction |
|  | cardiac complication |
|  | hypertensive heart disease |
|  | left ventricular dyssynchrony |

---

|  |  |
| --- | --- |
| Pathway | PPARG inhibits phenotypes assotiated with psoriasis |
|  | Neighbors of heart disease |

---

|  |  |
| --- | --- |
| MedScan ID | 9001650 |

---

|  |  |
| --- | --- |
| Alias | Cardiac event |
|  | Diseases, Cardiac |
|  | cardiac pathology |
|  | heart deterioration |
|  | cardiac valve deterioration |
|  | cardiac syndrome |
|  | Cardiac Disorder |
|  | cardiac deteriorations |
|  | pathogenesis of heart |
|  | heart deficiency |
|  | heart syndrome |
|  | deterioration of cardiac |
|  | heart dysfunctions |
|  | cardiac syndromes |
|  | deterioration of heart |
|  | cardiac disturbances |
|  | cardiac conducting system disease |
|  | deterioration of the myocardium |
|  | coronary event |
|  | Cardiopathy |
|  | Disorder of Heart |
|  | Heart Disorder |
|  | cardiac pathologies |
|  | atherosclerotic cardiac diseases |
|  | Heart Disease |
|  | ventricle deterioration |
|  | Cardiac events |
|  | atherosclerotic cardiac disease |
|  | pathology of heart |
|  | cardiac disturbance |
|  | heart pathology |
|  | cardiac pathogenesis |
|  | cardiac deterioration |
|  | heart dysfunction |
|  | coronary events |
|  | cardiac signs |
|  | Heart Disorders |
|  | pathogenesis of cardiac |
|  | heart pathologies |
|  | Diseases, Heart |
|  | Cardiac dysfunction |
|  | Cardiac dysfunctions |
|  | heart pathogenesis |
|  | Disease, Heart |
|  | Cardiac Diseases |
|  | disorders of the heart |
|  | pathologies of the heart |
|  | Heart Diseases |
|  | Cardiac Disease |
|  | myocardium deterioration |
|  | Morbus Cordis |
|  | Cardiac Disorders |
|  | Disease, Cardiac |
|  | heart deficiencies |
|  | cardiopathies |
|  | heart syndromes |
|  | myocardial deterioration |

---

|  |  |
| --- | --- |
| MeSH ID | D006331 |
|  | M0009951 |

---

|  |  |
| --- | --- |
| MeSH Heading | Heart Diseases |

---
